# Whole genome sequencing data of *Leptospira weilii* and *Leptospira kirschneri* isolated from human subjects of Sri Lanka

**DOI:** 10.1016/j.dib.2023.109840

**Published:** 2023-11-22

**Authors:** Indika Senavirathna, Dinesha Jayasundara, Janith Warnasekara, Michael A. Matthias, Joseph M. Vinetz, Suneth Agampodi

**Affiliations:** a Leptospirosis Research Laboratory, Department of Community Medicine, Faculty of Medicine and Allied Sciences, Rajarata University of Sri Lanka, Sri Lanka; b Department of Biochemistry, Faculty of Medicine and Allied Sciences, Rajarata University of Sri Lanka, Sri Lanka; c Department of Microbiology, Faculty of Medicine and Allied Sciences, Rajarata University of Sri Lanka, Sri Lanka; d Department of Community Medicine, Faculty of Medicine and Allied Sciences, Rajarata University of Sri Lanka, Sri Lanka; e Section of Infectious Disease, Department of Internal Medicine, School of Medicine, Yale University, New Haven, CT, USA; f International Vaccine Institute, Seoul, Republic of Korea; g Center for Public Health, Anuradhapura, Sri Lanka

**Keywords:** PacBio single molecule real-time, *Leptospira kirschneri*, *Leptospira weilii*, Whole genome, FMAS_RT1, Bio-sample, Sri Lanka, Leptospirosis

## Abstract

Leptospirosis is a re-emerging zoonotic disease. This article reports the complete genome sequences of three novel strains of Genus *Leptospira*: two from the species *Leptospira weilii* (FMAS_RT1, FMAS_PD2) and one from *Leptospira kirschneri* (FMAS_PN5). These isolates were recovered from the blood samples of acute febrile patients in different geographical and climatic zones of Sri Lanka. High-quality genomic DNA was extracted from the three isolates in mid-log phase cultures. Whole genome sequencing was conducted using the PacBio Single Molecule Real-Time (SMRT) platform to identify the species, genome features, and novelty of the strains. The annotation was conducted using RAST (Rapid Annotation Using Subsystem Technology version 2.0) and the NCBI Prokaryotic Genome Annotation Pipeline. The genome sequences of three isolates have been deposited in the Mendeley data repository and the National Center for Biotechnology Information (NCBI) repository. This data will be useful for future researchers when conducting comparative genomic analysis, revealing the exact mechanism of pathogenesis of leptospirosis and developing molecular diagnostic tools for early detection.

Specifications Table

**Table:** 

Subject	Infectious Disease
Specific subject area	Molecular data on leptospirosis
Type of data	Assembled and annotated draft genome of *Leptospira weilii*(02) and *Leptospira kirschneri*(01) Table Figure
How the data were acquired	Data were acquired through whole -genome sequencing which was performed using the PacBio Single Molecule Real-Time (SMRT) platform
Data format	Sequence files (FASTA) Raw Analysed
Description of data collection	Sequencing reads were generated by PacBio Single Molecule Real-Time (SMRT) platform. The obtain long reads were assembled using de novo Canu 2.1. A full Canu run protocol included three stages: correction, trimming, and assembly. The default parameters, as documented in the Canu documentation, were used in the above-mentioned process. The software Circlator was used to circularized the assemble genomes.
Data source location	Institution: Rajarata University of Sri Lanka City/Town/Region: Saliyapura, North Central Province Country: Sri Lanka
Data accessibility	Repository name: Mendeley Data Data identification number: 10.17632/3f8tvpw348.2 Direct URL to data: https://data.mendeley.com/datasets/3f8tvpw348/1 Repository name: NCBI Bio-project: PRJNA528695 NCBI Bio-Sample: SAMN11919596(FMAS_PN5) SAMN11919580(FMAS_PD2) SAMN11919575(FMAS_RT1) Direct URL to data: *Leptospira kirschneri* strain FMAS_PN5 https://www.ncbi.nlm.nih.gov/nuccore/CP092660.1 https://www.ncbi.nlm.nih.gov/nuccore/CP092657.1 https://www.ncbi.nlm.nih.gov/nuccore/CP092658.1 https://www.ncbi.nlm.nih.gov/nuccore/CP092659.1 https://www.ncbi.nlm.nih.gov/nuccore/CP092661.1 *Leptospira weilii* strain FMAS_PD2 https://www.ncbi.nlm.nih.gov/nuccore/CP092203.1 https://www.ncbi.nlm.nih.gov/nuccore/CP092202.1 https://www.ncbi.nlm.nih.gov/nuccore/CP092201.1 https://www.ncbi.nlm.nih.gov/nuccore/CP092204.1 https://www.ncbi.nlm.nih.gov/nuccore/CP092205.1 *Leptospira weilii* strain FMAS_RT1 https://www.ncbi.nlm.nih.gov/nuccore/CP092206.2 https://www.ncbi.nlm.nih.gov/nuccore/CP092207.2 https://www.ncbi.nlm.nih.gov/nuccore/CP092208.2 https://www.ncbi.nlm.nih.gov/nuccore/CP092209.2 https://www.ncbi.nlm.nih.gov/nuccore/CP092210.2

## 1 Value of the Data

• The limited availability of complete whole genome sequences for *Leptospira weilii* (3/23) and *Leptospira kirschneri* (3/38) in the NCBI database underscores the importance of utilizing whole genome sequencing for faster outbreak prediction and enhanced efficiency in the molecular epidemiology of infections, particularly during outbreaks.
• Researchers working on leptospirosis will benefit from this data in conducting studies related to vaccine development and revealing the molecular basis of pathogenesis in leptospirosis.
• Researchers focusing on specific genes (Virulence modifying genes) can conduct comparative genomic analysis and find evolutionary and contrasting changes depending on the geographical region and host.
• These datasets will also serve as a source for developing robust diagnostic assays for Leptospirosis*.*

## 2 Objective

The objective of this data set is to provide whole genome sequences of three new strains of *Leptospira* belonging to *Leptospira weilii* (FMAS_PD2 and FMAS_RT1) and *Leptospira kirschneri* (FMAS_PN5) recovered from patients in Sri Lanka (Peradeniya, Rathnapura, and Polonnaruwa).

## 3 Data Description

The whole genome data of the three isolates have been deposited in NCBI. General information on the strain, including the place of collection and the source of isolation, is contained in the ‘Bio-Sample SAMN11919596 (FMAS_PN5), SAMN11919580 (FMAS_PD2), SAMN11919575 (FMAS-RT1)’. The summary statistics for raw read data can be found in Table 2. Assembly information and general taxonomic information is included in “Bio-Project: PRJNA528695” Information about the assembly process, genome coverage, sequencing technology, genome annotation data (such as the number of genes, coding sequences, pseudo genes, etc. (Table 1), and the best-matching type-strain assembly is all included in the file titled “Assembly: GCA_024526015.1, GCA_022344045.1, GCA_022453935.2”. The genome annotation data are available in both NCBI Prokaryotic Genome Annotation Pipeline (PGAP) and RAST (Rapid Annotation using Subsystem Technology).

**Table 1:** Comparison of genome features of *Leptospira weilii* (2) and *Leptospira kirschneri* (1) with existing serovar.

Genome features	*Leptospira weilii* (FMAS_PD2)	*Leptospira weilii* (FMAS_RT1)	*Leptospira weilii* (CUD13)	*Leptospira kirschneri* (FMAS_PN5)	*Leptospira kirschneri serovar Cynopteri str. 3522 CT
Genome size (Mb)	4.9	5.1	4.36	4.9	4.4
GC content	40.5	40.5	40.9	36	36.3
Genes (total)	4508	4822	3934	4211	3637
CDSs (total)	4463	4777	3890	4167	3594
Genes (coding)	4204	4509	3687	3887	3339
CDSs (with protein)	4204	4509	3687	3887	3339
Genes (RNA)	45	45	44	44	43
rRNAs	1, 2, 2 (5S, 16S, 23S)	1, 2, 2 (5S, 16S, 23S)	1,2,2(5S, 16S, 23S)	1, 2, 2 (5S, 16S, 23S)	1,1,2(5S, 16S, 23S)
Complete rRNAs	1, 2, 2 (5S, 16S, 23S)	1, 2, 2 (5S, 16S, 23S)	1, 2, 1,2,2 (5S, 16S, 23S)	1, 2, 2 (5S, 16S, 23S)	1,1,2 1,1,2(5S, 16S, 23S)
tRNAs	38	38	37	37	37
ncRNAs	2	2	2	2	2
Pseudo Genes (total)	259	268	203	280	195
CDSs (without protein)	259	268	203	280	195
Pseudo Genes (ambiguous residues)	0 of 259	0 of 268	0 of 203	0 of 280	0 of 195
Pseudo Genes (frame shifted)	174 of 259	182 of 268	145 of 203	160 of 280	144 of 195
Pseudo Genes (incomplete)	143 of 259	159 of 268	118 of 203	167 of 280	80 of 195
Pseudo Genes (internal stop)	40 of 259	40 of 268	36 of 203	61 of 280	32 of 195
Pseudo Genes (multiple problems)	83 of 259	94 of 268	80 of 203	93 of 280	54 of 195
CRISPR Arrays	2	2	2	8	2

⁎ Reference genome-*Leptospira kirschneri serovar Cynopteri str. 3522 CT*.

**Table 2:** PacBio_MD, raw read data summary statistics.

	FMAS_RT1	FMAS_PD2	FMAS_PN5
Main genome scaffold total:	993,618	1,092,594	652,429
Main genome contig total:	993,618	1,092,594	652,429
Main genome scaffold sequence total:	8044.115 MB	8879.706 MB	5575.934 MB
Main genome contig sequence total:	8044.115 MB	8879.706 MB	5575.934 MB
Main genome scaffold N/L50:	316,725/8.981 KB	346,377/9.005 KB	207,812/9.48 KB
Main genome contig N/L50:	316,725/8.981 KB	346,377/9.005 KB	207,812/9.48 KB
Main genome scaffold N/L90:	134,525/11.962 KB	128,295/12.548 KB	144,461/10.826 KB
Main genome contig N/L90:	134,525/11.962 KB	128,295/12.548 KB	144,461/10.826 KB
Max scaffold length:	273.279 KB	240.562 KB	221.54 KB
Max contig length:	273.279 KB	240.562 KB	221.54 KB
Number of scaffolds > 50 KB:	854	966	685
% main genome in scaffolds > 50 KB:	0.85 %	0.86 %	0.98 %

**Fig. 1:**
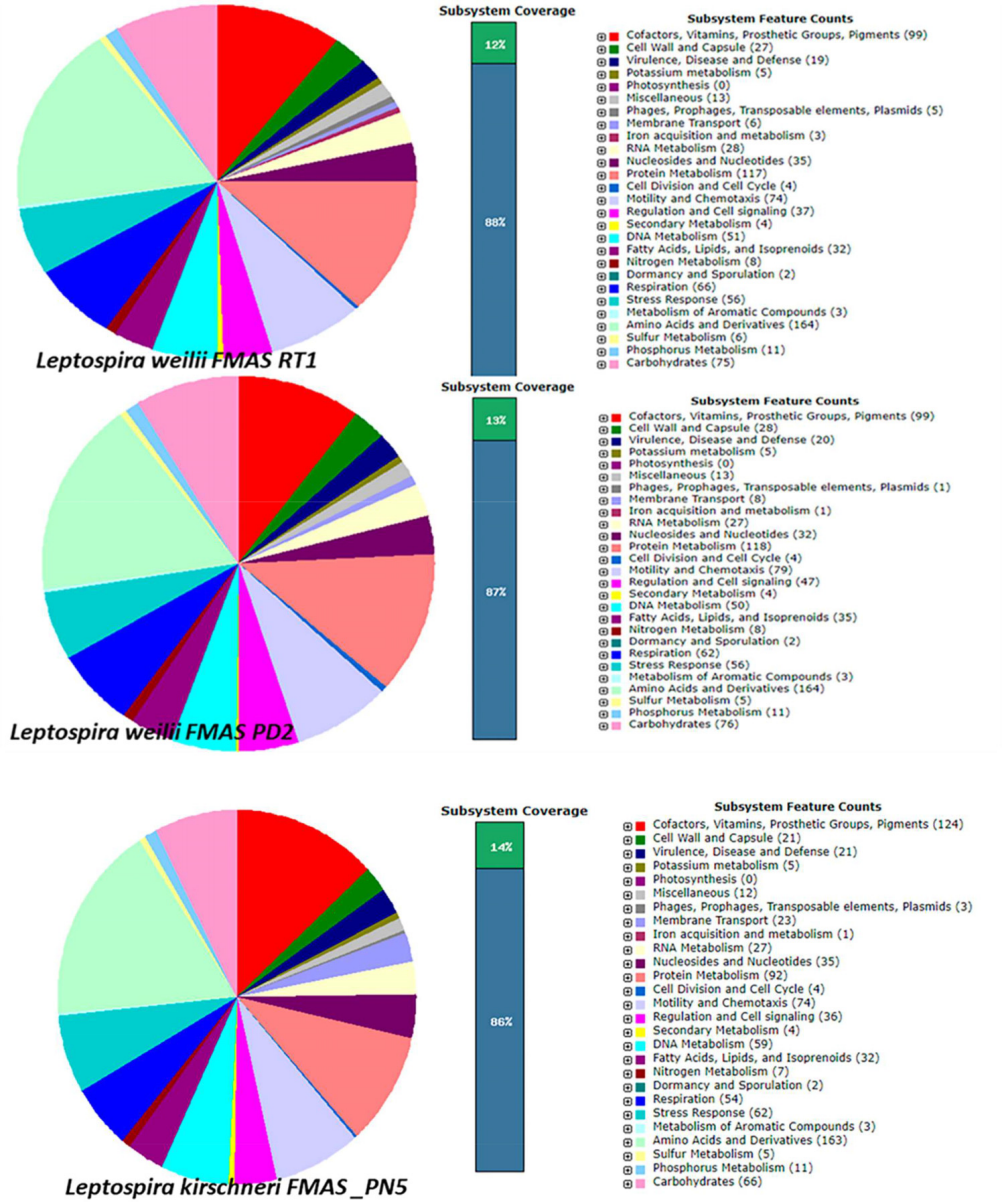
The Rapid Annotation using Subsystem Technology (RAST) server's annotation was used to create the pie-chart showing the distribution of subsystem categories for the strains *Leptospira weilii*-FMAS_RT1, FMAS_PD2 and *Leptospira krischneri* FMAS_PN5 which was then viewed in the SEED Viewer. The percentage of total proteins encoded in the genomes of strains FMAS_RT1 and FMAS_PD2 that could or could not be allocated to subsystem categories are shown by the green and blue bars in the bar diagram on the left, respectively. Green color box indicates the coding percentage for subsystem. Blue color box indicates the coding percentage not in the subsystem.

**Fig 2:**
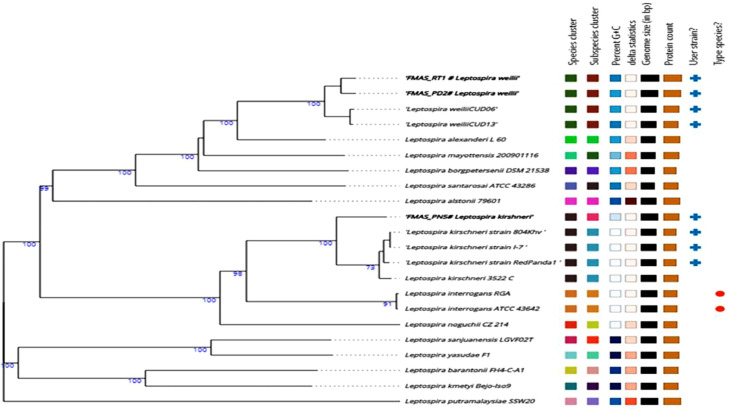
Tree inferred with FastME 2.1.6.1 (15) from GBDP distances calculated from genome sequences. The branch lengths are scaled in terms of GBDP distance formula *d5*. The numbers above branches are GBDP pseudo-bootstrap support values > 60 % from 100 replications, with an average branch support of 56.2 %. The tree was rooted at the midpoint(16). If the organism belongs to the same species cluster or subspecies cluster, the percentage G + C delta statistics are indicated by the same color. The length of the black and brown boxes indicates the genome size and protein count.

**Fig. 3:**
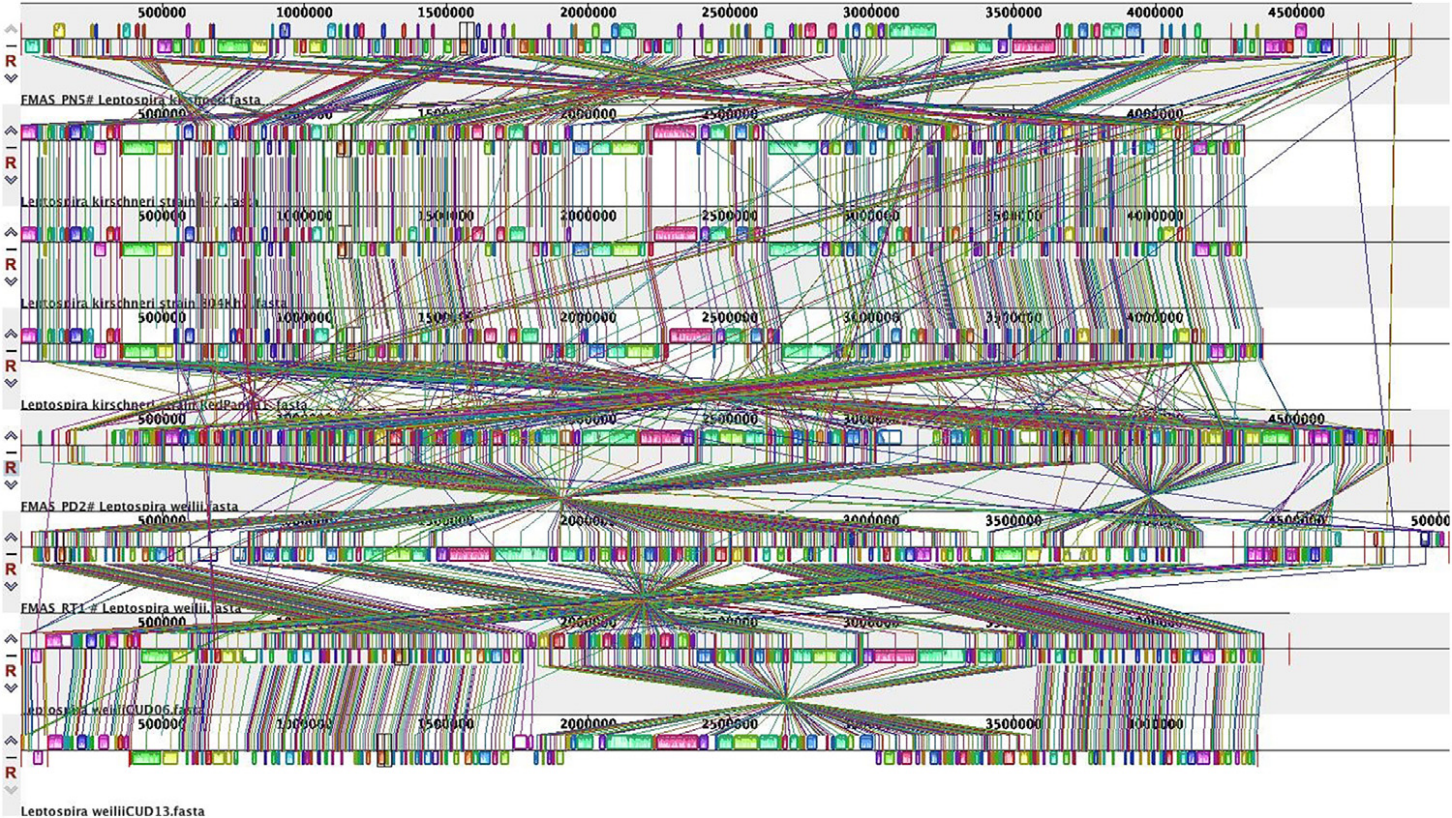
Multiple alignments using Mauve 2. Snap shot of the Genome comparison of three Sri Lankan isolates (*L. weilii* and *L. kirschneri*) and the other available genomes. The strains were aligned and arranged using Mauve genome aligner in the following order: Top FMAS_PN5, *Leptospira kirschneri* strain I-7, *Leptospira kirschneri* strain 804Khv, *Leptospira kirschneri* strain RedPanda1 FMAS_PD2, FMAS_RT1, *Leptospira weilii* CUD06 and *Leptospira weilii* CUD13 Large collinear blocks (LCBs) correspond mainly to conserved synteny regions, as represented by colored boxes. The lines between the genomes connect the blocks that are conserved between two strains, indicating rearrangements.

The genome annotation of *Leptospira weilii* (FMAS_PD2*), Leptospira weilii* (FMAS_RT1) and *Leptospira kirschneri* (FMAS-PN5) using Rapid Annotation Subsystem Technology (RAST) server resulted in a total number of subsystems 231, 230 and 232 respectively (Fig. 1). Most of the subsystem features (annotated genes) were related to amino acids and derivatives (164 and 163genes); followed by protein metabolism (FMAS-RT1–117, FMAS_PD2–118) cofactors, vitamins, prosthetic groups, pigments (FMAS_PN5–124). Some interesting subsystems to further explore from the annotation include genes coding for virulence, pathogenicity, and defense. Interestingly, pathogenicity islands (FMAS_PD2), phage baseplate proteins and phage packaging machinery genes (FMAS-RT1) and phage baseplate protein gene (1) and phage tail fiber protein (1) were observed (FMAS_PN5) in the three isolates. To visualize the organization of the genome and discover potential genome rearrangements among strains, conserved regions were visualized using a Mauve genome aligner. Large Collinear Blocks (LCBs) were identified in different ways. Colored rectangular and variant-specific regions (genomic islands, GI) or white region spaces within or between LCBs were identified in both chromosomes in all strains. Massive genomic rearrangements were observed in Sri Lankan strains compared to other strains belonging to respective species (Fig. 3). Based on phylogenetic analysis, FMAS_PD2 and FMAS_RT1 were found to be closely related to *Leptospira weilii* CUD06 and CUD07. However, FMAS_PN5 belongs to a separate sub-clade that is distinct from the main *Leptospira krischneri* cluster (Fig. 2).

## 4 Experimental Design, Materials and Methods

### 4.1 Bacteria isolation

We received culture samples from febrile patients clinically suspected of leptospirosis from a large ongoing clinical study. The blood samples were gathered from patients either upon admission to the ward or when they presented themselves at the outpatient department (OPD). A total of 7 mL of blood was drawn, and at the bedside, inoculation was carried out by applying 2–4 drops (equivalent to 100–500 µL) into two tubes, each containing 9 mL of EMJH semisolid media supplemented with antibiotics (5-fluorouracil and neomycin). The blood samples combined with EMJH media for cultivation were then placed in an incubator set at 30 °C for incubation [1], A culture collection was conducted involving three categories of patients. This included individuals who had acute undifferentiated fevers (with temperatures exceeding 38 °C) and sought medical care in the adult wards (aged over 13 years) of Teaching Hospital Anuradhapura(THA), THP, Provincial General Hospital Rathnapura (PGHR), as well as Base Hospital Avissawella (BHA), encompassing both outpatients and those hospitalized, who were considered potential cases of leptospirosis [2], and clinical details [3] are published elsewhere. *Leptospira weilii* strains, FMAS_PD2 and FMAS_RT1, were isolated from samples collected in Peradeniya and Rathnapura, and *Leptospira kirschneri* FMAS_PN5 was isolated from samples collected in Polonnaruwa, Sri Lanka. These three strains had only a few (>10) passages before genomic DNA extraction for WGS. Organisms maintained in semisolid EMJH media were sub-cultured into liquid EMJH medium. Fresh samples were acquired from log phase growth [1].

### 4.2 DNA extraction sequencing and de novo assembly

Five (5 mL samples from a growing culture (mid-log phase) were centrifuged at 3500 rpm, and the supernatant was discarded. The pellets were then washed three to four times using phosphate-buffered saline (PBS). The DNeasy Blood & Tissue Kit from Qiagen was used to extract DNA using a Gram-negative bacteria protocol that included an RNase cleanup step after proteinase *K* + Buffer ATL incubation. The amount of each of the total extracted DNA was measured with a Qubit 4 fluorometer. From extracted high-quality gDNA, multiplexed PacBio Single Molecule RealTime (SMRT)bell libraries were constructed using the SMRTbell^Ⓡ^ Express Template Prep Kit. 2.0. g-tubes TM from Covaris (Woburn, MA, USA) was used to shear 1 g of genomic DNA, and AMPure PB beads from Pacific Bioscience were used to concentrate the DNA. The DNA was finally repaired following an overnight ligation to an affixed barcoded 8A adaptor (Pacific Bioscience) [4,5]. The size selection instructions provided by the manufacturer, Sage Science, Beverly, Massachusetts, USA, for Blue Pippin TM 4 kb or more were adhered to. Purified DNA purification and annealing of the sequencing primer to the SMRTbell TM template were examined using an RS Remote (Pacific Biosciences) calculator. At least 600× of read coverage was attained for the three isolates. Raw read data was preprocessed using an internal quality control pipeline. The data quality of the raw reads was tested and quality checked using LRPlot v0.5.2. The genomes were assembled with Canu 2.1 using default settings given in the documentation [6] and Circlator [7] and then circularized.

### 4.3 Genome annotation

Genome annotation was conducted using RAST (Rapid Annotation Using Subsystem Technology version 2.0) [8] (https://rast.nmpdr.org/) and NCBI Prokaryotic Genome Annotation Pipeline was used to annotate the draft genome [9].

### 4.4 Determination of closely related type strains

The determination of the closest type strain genomes was carried out using two complementary methods. Firstly, the MASH algorithm was used to compare all user genomes with the type strain genomes available in the TYGS database, selecting the ten type strains with the smallest MASH distances [10]. Secondly, 16S rDNA gene sequences were extracted from the user genomes using RNAmmer [11] and compared against the 16S rDNA gene sequences of the 16,578 type strains in the TYGS database using BLAST. The top 50 matching type strains for each user genome were identified based on the bitscore, and precise distances were calculated using the GBDP approach with the 'coverage' algorithm and distance formula d5 [11], [12], [13]. These distances were then utilized to determine the ten closest type strain genomes for each user genome.

The phylogenomic inference involved pairwise comparisons of genomes using GBDP, which yielded accurate inter genomic distances [12]. Digital DDH values and confidence intervals were determined using GGDC 3.0 [12,14]. A balanced minimum evolution tree with branch support was constructed using FASTME 2.1.6.1, incorporating SPR post processing [15], [16], [17]. Type-based species clustering was performed using a 70 % dDDH radius around 13 type strains. Subspecies clustering utilized a 79 % dDDH threshold [18,19]. The results are presented in Table 1 and 4 Mendeley (https://data.mendeley.com/datasets/3f8tvpw348/1). The Mauve aligner was utilized to identify matches and organize them into locally collinear blocks (LCBs) [20].

## Ethics Statements

Witten informed consent was obtained from the entire participants in this study. Ethical clearance for this study was obtained from the Ethics Review Committee of the Faculty of Medicine and Allied Sciences, Rajarata University of Sri Lanka. Protocol No. ERC/2015/18.

## CRediT authorship contribution statement

**Indika Senavirathna:** Conceptualization, Methodology, Software, Validation, Data curation, Writing – original draft, Visualization, Investigation, Visualization, Investigation. **Dinesha Jayasundara:** Writing – original draft, Visualization, Investigation, Supervision, Software, Validation. **Janith Warnasekara:** Visualization, Investigation. **Michael A. Matthias:** Supervision, Software, Validation. **Joseph M. Vinetz:** Supervision, Software, Validation. **Suneth Agampodi:** Conceptualization, Methodology, Software, Validation, Supervision, Software, Validation, Supervision, Software, Validation.

## Data Availability

Whole genome sequence data of Leptospira weilii and Leptospira Leptospira kirschneri isolated from human subjects Sri Lanka (Original data) (Mendeley Data)
